# Impact of environmental asymmetry on epithelial morphogenesis

**DOI:** 10.1038/s41598-022-15343-y

**Published:** 2022-07-05

**Authors:** Kentaro Morikawa, Daichi Kuroda, Yasuhiro Inoue

**Affiliations:** Department of Micro Engineering, Graduate School of Engineering, Kyoto University, Kyoto, Japan

**Keywords:** Computational biophysics, Morphogenesis, Computational models

## Abstract

Epithelial folding is a universal biological phenomenon in morphogenesis, typical examples being brain gyri, villi of the intestinal tract, and imaginal discs in invertebrates. During epithelial morphogenesis, the physical constraints imposed by the surrounding microenvironment on epithelial tissue play critical roles in folding morphology. In this study, we focused on the asymmetry of the environmental constraints sandwiching the epithelial sheet and introduced the degree of asymmetry, which indicates whether the basal or apical side of the epithelium is closer to the constraint wall. Then, we investigated the relationship between the degree of asymmetry and epithelial folding morphology using three-dimensional vertex simulations. The results show that the folding patterns of the epithelial sheets change from spot patterns to labyrinth patterns and then to hole patterns as the degree of asymmetry changes. Furthermore, we examined the pattern formation in terms of the equation of out-of-plane displacement of the sheet derived from the mechanical energy functional.

## Introduction

Epithelial tissues consist of single- or multilayered sheets of cells that cover the external surface of animals and the internal surface of visceral organs. The epithelial morphology is characterized by undulating shapes and is formed by the complex folding of the epithelial sheet during morphogenesis, e.g. the formation of brain gyri^1^, villi of the intestinal tract^2,3^, sea urchin archenterons^4^, leg and wing imaginal discs of *Drosophila melanogaster*^5,6^, horn primordia of beetles^7,8^, and helmet primordia of treehoppers^9^. Thus, epithelial folding plays a major role in morphogenesis. Folded epithelial structures serve specific biological functions. The surface area of neocortex is a critical determinant of intellectual ability^10^ and the folding shape enables the mammalian brain to expand its surface area in the skull^11^. The lumen of the intestinal tract is covered by villi composed of a single layer of epithelial cells, which provide an abundant surface area for nutrient absorption^2,3^. Furthermore, the imaginal disc of insect exoskeleton is the folding structure in which the completed shape of the exoskeleton is coded and is stored in the small body of the larva^5–9^. Epithelial folding is often restricted by the physical environment. In brain or intestine morphogenesis, there is a substratum layer that is different in elasticity from the epithelial layer^1,2^ and the difference causes the buckling patterns^12,13^. In *Drosophila* imaginal discs, the peripodial membrane adhering to one side of the epithelial tissue influences morphology^4,5^. And in the beetle horn primordium, outward deformation is constrained by the hard cuticle capsule of the larva^8^. These examples represent the asymmetric properties of the epithelial layer environment, suggesting that they may have a critical impact on pattern formation. Regarding pattern formation of thin films with environmental constraints, experiments using spherically shaped elastic bilayer materials with a thicker inner layer inside the thin film have shown that the ratio of the inner layer thickness to the thin film thickness determines the transition between the labyrinth and hexagonal phases^14^. This pattern formation is theoretically analyzed as the dynamical system described by the Swift-Hohenberg-like equation. The Swift–Hohenberg equation^15^ was first proposed as a model for the Rayleigh–Benard convection. Similar models have subsequently appeared in models of various physical phenomena^16^, such as granular materials^17^, self-assembled nanoparticles^18^, and the fiber laser^19^.

The three-dimensional (3D) vertex model is employed to simulate the morphological dynamics of epithelial morphogenesis^20–25^. In this model, each cell is represented as a polyhedron, and the epithelial sheet is modeled as a network of the vertices and edges of the constituent cellular polyhedrons. Several versions of the vertex model have been developed to respond to the needs of the application. In this study, we adopted a model that can deal with cell rearrangement, large-scale tissue deformation, and cell proliferation^26,27^.

The purpose of this study was to investigate the impact of environmental constraint on epithelial folding patterns, with a focus on environmental asymmetry. To achieve this, we developed a mathematical model to describe the mechanical interaction with an asymmetric environment. In this model, a hypothetical epithelial monolayer sheet was sandwiched between parallel elastic walls constraining the out-of-plane displacement of the sheet. The degree of environmental asymmetry was represented by different combinations of wall-to-sheet distances on the apical and basal sides of the epithelial sheet.

## Methods

### Mathematical models for simulating multicellular dynamics

In the 3D vertex model, a cell is represented as a polyhedron. Because epithelial tissue comprises a group of similar cells closely attached to their neighbors, it can be modeled as a network of connected polyhedrons whose vertices and edges are shared by adjacent ones. The kinematics of tissue sheet deformation can be described based on the locations and movements of the vertices of individual polyhedral units constituting the sheet. The movement of the *i*-th vertex can be described by

1 $$\begin{array}{*{20}c} {\eta \left( {\frac{{d{\varvec{r}}_{i} }}{dt} - {\varvec{v}}_{i}^{{{\text{loc}}}} } \right) = - \nabla_{i} U - \eta V,} \\ \end{array}$$

where ${\varvec{r}}_{i} \user2{ }$ represents its position vector, ${\varvec{v}}_{i}^{{{\text{loc}}}}$ represents the local velocity vector determined from its current location and those of its neighboring vertices, $\eta$ is the friction coefficient, $U$ is the potential energy function, and ${\varvec{V}}$ represents the velocity of the system’s center of gravity (CoG). Based on previous studies^23–25,28^, the local velocity vector ${\varvec{v}}_{i}^{{{\text{loc}}}}$ given in Eq. (1) can be defined as the mean velocity vector of the surrounding vertices:

2 $$\begin{array}{*{20}c} {{\varvec{v}}_{i}^{{{\text{loc}}}} = \frac{1}{{1 + \mathop \sum \nolimits_{j}^{{{\text{vertex}}}} \chi_{{V_{i} }} \left( j \right)}}\left( {\frac{{d{\varvec{r}}_{i} }}{dt} + \mathop \sum \limits_{j}^{{{\text{vertex}}}} \frac{{d{\varvec{r}}_{i} }}{dt}\chi_{{V_{i} }} \left( j \right)} \right),} \\ \end{array}$$

where $\chi_{{V_{i} }} \left( j \right)$ is the indicator function for a subset $V_{i}$ that is the set of all vertices directly connected to the $i$-th vertex by edges. Here, the number of vertices connected to vertex $i$ is expressed as $\sum\nolimits_{j}^{{{\text{vertex}}}} {\chi_{{V_{i} }} } \left( j \right).$ In our simulations, this sum for vertex $i$ is always equal to four because the tissues are monolayers and there are three cells surrounding a vertex. This local velocity vector is introduced to satisfy Galilean invariance^28^. There is no noise term in this vertex motion, but there is randomness in cell growth, described below, which is reflected in the equation of motion through the energy function. During morphogenesis, tissue deformation occurs over a longer timeframe than during cell displacements. Therefore, Eq. (1) neglects the effects of inertia on cellular dynamics and predominantly accounts for the effects of viscosity.

**Figure 1:**
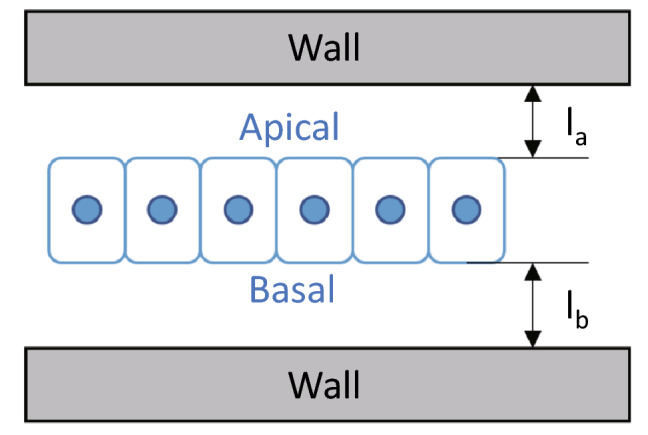
Schematic diagram of epithelial tissue and the elastic wall that provides physical constraint to simulate cell proliferation dynamics in our model. The elastic walls are located at the apical and basal sides of the epithelium, and the distance between them is denoted by $l_{{\text{a}}}$ and $l_{{\text{b}}}$, respectively.

Vertex behavior defined by Eq. (1) depends on the potential energy function. This study assumes that individual cells have the following types of potential energy: volume elasticity energy $U^{{{\text{VE}}}}$, surface elasticity energy $U^{{{\text{SE}}}}$, height elasticity energy $U^{{{\text{HE}}}}$, and environmental constraint energy $U^{{{\text{EN}}}}$. To account for environmental effects, the tissue kinematics model includes two elastic walls lying parallel to the tissue sheet, one at a distance of $l_{{\text{a}}}$ from the apical surface and the other at a distance of $l_{{\text{b}}}$ from the basal surface (Fig. 1). The total energy function $U$ is defined as follows:

3 $$\begin{array}{*{20}c} {U = U^{{{\text{VE}}}} + U^{{{\text{SE}}}} + U^{{{\text{HE}}}} + U^{{{\text{EN}}}} .} \\ \end{array}$$

Each of these types of energy is defined as follows:

4 $$\begin{array}{*{20}c} {U^{{{\text{VE}}}} = \mathop \sum \limits_{i}^{{{\text{cell}}}} \frac{1}{2}k_{{\text{V}}} \left( {\frac{{V_{i}^{{\text{c}}} }}{{V^{{{\text{c}},{\text{eq}}}} }} - 1} \right)^{2} ,} \\ \end{array}$$

5 $$\begin{array}{*{20}c} {U^{{{\text{SE}}}} = \mathop \sum \limits_{i}^{{{\text{cell}}}} \frac{1}{2}k_{{\text{S}}} \left( {\frac{{S_{i}^{{\text{c}}} }}{{S^{{{\text{c}},{\text{eq}}}} }} - 1} \right)^{2} ,} \\ \end{array}$$

6 $$\begin{array}{*{20}c} {U^{{{\text{HE}}}} = \mathop \sum \limits_{i}^{{{\text{cell}}}} \frac{1}{2}k_{{\text{H}}} \left( {\frac{{H_{i}^{{\text{c}}} }}{{H^{{{\text{c}},{\text{eq}}}} }} - 1} \right)^{2} ,} \\ \end{array}$$

7 $$\begin{array}{*{20}c} {U^{{{\text{EN}}}} = \mathop \sum \limits_{i}^{{{\text{cell}}}} \frac{1}{2}k_{{{\text{EN}}_{i} }}^{{{\text{api}}}} \left( {l_{i}^{{{\text{api}}}} - l_{{\text{a}}} } \right)^{2} + \mathop \sum \limits_{i}^{{{\text{cell}}}} \frac{1}{2}k_{{{\text{EN}}_{i} }}^{{{\text{bsl}}}} \left( {l_{i}^{{{\text{bsl}}}} - l_{{\text{b}}} } \right)^{2} ,} \\ \end{array}$$

where *Ʃ*_*i*_^cell^ represents summation across all cells, and $l_{i}^{{{\text{api}}}}$ and $l_{i}^{{{\text{bsl}}}}$ represent the out-of-plane displacement of the center of gravity of the *i*-th cell from the apical and basal surfaces, respectively. Moreover, $k_{{{\text{EN}}_{i} }}^{{{\text{api}}}}$ and $k_{{{\text{EN}}_{i} }}^{{{\text{bsl}}}}$ indicate the elasticity coefficients of the wall on the apical and basal sides, respectively. These variables are defined as follows:

8 $$\begin{array}{*{20}c} {k_{{{\text{EN}}_{i} }}^{{{\text{api}}}} = \left\{ {\begin{array}{*{20}c} {k_{{{\text{EN}}}} S_{i}^{{{\text{api}}}} \left( {l_{i}^{{{\text{api}}}} > l_{{\text{a}}} } \right)} \\ {0 \left( {l_{i}^{{{\text{api}}}} \le l_{{\text{a}}} } \right)} \\ \end{array} } \right., k_{{{\text{EN}}_{i} }}^{{{\text{bsl}}}} = \left\{ {\begin{array}{*{20}c} {k_{{{\text{EN}}}} S_{i}^{{{\text{bsl}}}} \left( {l_{i}^{{{\text{bsl}}}} > l_{{\text{b}}} } \right)} \\ {0 \left( {l_{i}^{{{\text{bsl}}}} \le l_{{\text{b}}} } \right)} \\ \end{array} } \right.,} \\ \end{array}$$

where $k_{{{\text{EN}}}}$ represents the characteristic elasticity coefficient of the wall, and $S_{i}^{{{\text{api}}}}$ and $S_{i}^{{{\text{bsl}}}}$ represent the apical and basal surface areas of the $i$-th cell, respectively. In this study, tissue growth is represented by the cell proliferation model^27^. Cell division is represented by the division of polyhedral, and cell growth is represented by changes in $V^{{{\text{c}},{\text{ eq}}}}$ and corresponding changes in $S^{{{\text{c}},{\text{eq}}}}$ ($H^{{{\text{c}},{\text{eq}}}}$ is set constant). Each cell is assumed to divide along the long axis of the cross sectional cell shape normal to apicobasal axis. Each cell is constrained to adopt the shape of prism and to always have a basal and apical side. The cell cycle is represented by the mean cycle period $\tau^{{{\text{cycle}}}}$ and its standard deviation $\sigma^{{{\text{cycle}}}}$. The percentages of the duration of G1, S, G2, and M phases within the cell cycle are set as ${\Psi }^{{\text{I}}}$, ${\Psi }^{{{\text{II}}}}$, ${\Psi }^{{{\text{III}}}}$ and ${\Psi }^{{{\text{IV}}}}$, respectively. This model does not take into account cell removal in this simulation. Table 1 lists all model constants used in this study.

**Table 1:** Model constants.

Symbol	Value	Description
*η*	0.25	Friction coefficient of vertices
*k*_V_	20.0	Constant of cell volume elasticity
*k*_S_	0.256	Constant of cell surface elasticity
*k*_H_	0.1	Constant of cell height elasticity
*k*_CL_	40.0	Constant of surface collision
*k*_EN_	0.01	Characteristic constant of constraint of elastic walls elasticity
*V*^c,eq^	1.0	Cell volume at stress free state
*S*^c,eq^	$\frac{{2V^{{{\text{c}},{\text{eq}}}} }}{{H^{{{\text{c}},{\text{eq}}}} }} + \sqrt {8\sqrt 3 V^{{{\text{c}},{\text{eq}}}} H^{{{\text{c}},{\text{eq}}}} }$	Cell surface area (hexagonal prism) at stress free state
*H*^c,eq^	1.0	Cell height at stress free state
*σ*	1.0	Threshold length of surface collision
*τ*^cycle^	1000	Statistical average of cell cycle
*σ*^cycle^	10	Standard deviation of cell cycle
Δ*t*	0.0002	Time step size for numerical integration of Eq. (1)
${\Psi }^{{\text{I}}}$	60	Percentage of G1 phase in the cell cycle
${\Psi }^{{{\text{II}}}}$	20	Percentage of S phase in the cell cycle
${\Psi }^{{{\text{III}}}}$	10	Percentage of G2 phase in the cell cycle
${\Psi }^{{{\text{IV}}}}$	10	Percentage of M phase in the cell cycle

Focusing on the folding structures induced by cell proliferation, we used a flat, homogeneous epithelial monolayer sheet for the initial condition. In the initial state, the tissue consists of 40 × 40 hexagonal prism shaped cells aligned in a regular hexagonal lattice. The model constants are set so that the initial state is a steady state without cell proliferation. In order to analyze the folding structure, we performed cell proliferation simulation under the periodic boundary conditions at the edges of the tissue.

## Results

### Proximity of the elastic wall and peak-to-peak folding distance

This section presents the results of the 3D sheet morphology simulations obtained by altering the degree of constraint on the out-of-surface deformation. In this run, the same degrees of constraint were assumed on both sides of the epithelial sheet. Specifically, each of the following values was entered simultaneously into both $l_{{\text{a}}}$ and $l_{{\text{b}}}$ in Eq. (7): 0.20, 0.30, 0.40, 0.50, 1.0, 1.5, and 2.0.

**Figure 2:**
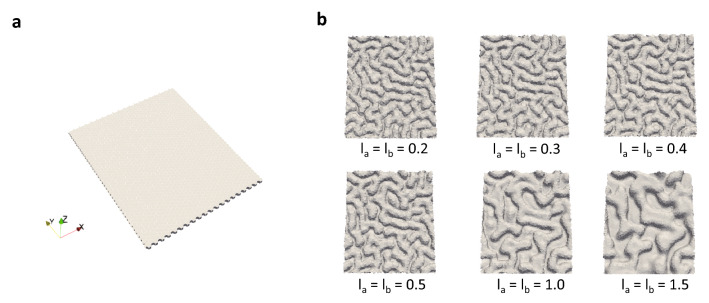
The initial condition and the results of the epithelial folding simulations. (**a**) Monolayer sheet as the initial condition. (**b**) Snapshots of epithelial folding simulated under the conditions of $l_{{\text{a}}} = l_{{\text{b}}} = 0.2, 0.3, 0.4, 0.5, 1.0, 1.5$. All snapshots show the simulation results at time $t = 0.5\tau_{{{\text{avg}}}}^{{{\text{cycle}}}} .$ The closer the distance between the epithelial sheet and the elastic wall, the smaller the peak-to-peak distance of folding.

The initial shape of the epithelial sheet and the results of the simulations are presented graphically in Fig. 2. The initial shape is planar as shown in Fig. 2a. As cells proliferate under periodic boundary conditions, buckling of the tissue is induced. This causes the out-of-plane deformed epithelial tissue to collide with the wall. This process is shown in Supplementary Video 1. The sequence in Fig. 2b corresponds to the order of proximity between the elastic wall and the epithelial sheet (i.e., 0.20–1.5). Closer proximity of the elastic wall resulted in a smaller peak-to-peak folding distance.

### The degree of asymmetry relative to the *XY* plane and peak-to-peak folding distance

The simulation results shown in the previous section represent the cases where the same degrees of environmental constraint were applied to the apical and basal sides of the sheet (i.e., *l*_a_ = *l*_b_). In this study, we evaluated the impact of asymmetric environmental constraint on the folding patterns by changing the degree of asymmetry relative to the *xy* plane. For this purpose, two variables were introduced: the total distance of the elastic walls from the epithelial sheet *l*_sum_ (= *l*_a_ + *l*_b_) and the degree of asymmetry relative to the *xy* plane Λ (= *l*_b_*/l*_sum_). Specifically, *l*_sum_ had one of the following values: 0.60, 1.0, 1.4, 2.0, or 3.0. For each *l*_sum_ value, the asymmetry indicator Λ ranged from 0 to 1.

**Figure 3:**
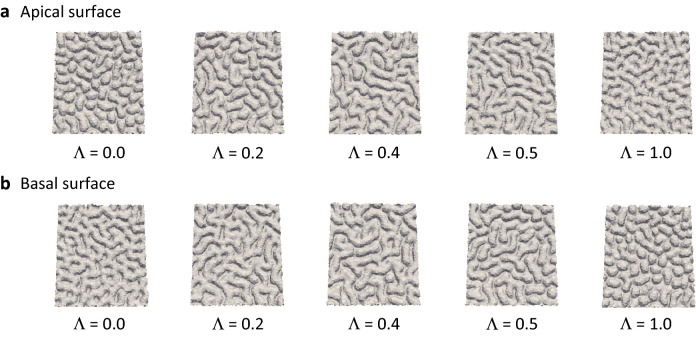
Snapshots of epithelial folding simulated under the conditions of ${\Lambda } = 0.0, 0.2, 0.4, 0.5, 1.0$. (**a**) Apical surface. (**b**) Basal surface. All snapshots show the simulation results at time $t = 0.5\tau_{{{\text{avg}}}}^{{{\text{cycle}}}} , l_{{{\text{sum}}}} = 1.0$. The ridges on the apical surface are shorter when the degree of asymmetry ${\Lambda }$ is small (the basal side is closer to the elastic wall), and the length of ridges increases as ${\Lambda }$ increases. The reverse occurs with the folding patterns of the basal surface: the ridges become shorter as ${\Lambda }$ becomes larger.

The representative 3D morphology of the apical surface simulated using *l*_sum_ = 1.0 is illustrated graphically using the emboss effect in Fig. 3a; the sequence represents increasing values of Λ (i.e., 0–1.0). Similarly, the 3D structures of the basal surface simulated using *l*_sum_ = 1.0 are presented graphically in Fig. 3b, where the sequence represents increasing values of Λ (i.e., 0–1.0). These results demonstrate that increases in Λ from 0 to 0.5 (i.e., from maximum asymmetry to symmetry) resulted in longer (and narrower) folds. Moreover, the folding patterns were similar between the apical side at Λ = *p* (*p* representing a given value between 0 and 1) and the basal side at Λ = 1 − *p*, supporting the mathematical validity of the model.

### Environmental constraints and wavenumbers of folds

The previous section showed that the presence of elastic walls in the sheet kinematics model induced folding of the growing tissue (i.e., emergence of grooves and ridges), and that the folding patterns were dependent on the distances between the walls and the apical and basal surfaces. These findings allow further mathematical considerations of epithelial morphogenesis. In this section, we introduce an indicator for the peak-to-peak folding distance and provide a detailed quantitative analysis of this relationship.

The folding distance indicator *I*(*u*_*x*_*,u*_*y*_) is derived as follows. First, the data regarding the vertices and CoGs of the cells on the apical surface were extracted from the simulation results described above. The area of computation was divided into unit grid cells of the same size, the total number of which was *N*_*x*_ × *N*_*y*_, with *N*_*x*_ and *N*_*y*_ denoting the number of unit grid cells on the *x* and *y* axes, respectively. Here, *N*_*x*_ and *N*_*y*_ were set to 35. The *z*-axis coordinates of the vertices and CoGs included in a given grid cell were determined, and their average value was defined to represent the out-of-surface displacement. A discrete second-order displacement field *z*(*x,y*) was derived by linking the displacements and coordinates (*x,y*) of the center of the individual unit grid cells. A discrete Fourier transform of the displacement field *z*(*x,y*) and its power spectrum *I* can be expressed as follows:

9 $$\begin{array}{*{20}c} {F\left( {\widetilde{{u_{x} }}, \widetilde{{u_{y} }}} \right) = \mathop \sum \limits_{x = 0}^{{N_{x} - 1}} \mathop \sum \limits_{y = 0}^{{N_{y} - 1}} z\left( {x, y} \right)e^{{ - 2\pi i\left( {\frac{{\widetilde{{u_{x} }}x}}{{N_{x} }} + \frac{{\widetilde{{u_{y} }}y}}{{N_{y} }}} \right)}} ,} \\ \end{array}$$

10 $$\begin{array}{*{20}c} {I\left( {\widetilde{{u_{x} }},\widetilde{{u_{y} }}} \right) = \left| {F\left( {\widetilde{{u_{x} }}, \widetilde{{u_{y} }}} \right)} \right|^{2} ,} \\ \end{array}$$

where $\widetilde{{u_{x} }}$ and $\widetilde{{u_{y} }}$ represent the wavenumbers over the system’s $x$- and $y$-axis lengths, $X$ and $Y$, respectively. The wavenumbers $\widetilde{{u_{x} }}$ and $\widetilde{{u_{y} }}$ were normalized to the characteristic length of the system $L = \sqrt {X^{2} + Y^{2} }$, and $u_{x}$ and $u_{y}$ were defined as follows:

11 $$\begin{array}{*{20}c} {u_{x} = \frac{L}{X}\widetilde{{u_{x} }},} \\ \end{array}$$

12 $$\begin{array}{*{20}c} {u_{y} = \frac{L}{Y}\widetilde{{u_{y} }}.} \\ \end{array}$$

The distribution of the power spectrum *I*(*u*_*x*_*,u*_*y*_) thus obtained was used as an indicator of the peak-to-peak folding distance. A study revealed that the distribution of the power spectrum reflects the peak-to-peak folding distance and the orientation of the folds, demonstrating its utility for analyzing folding distance^25^.

Moreover, on the basis of the distribution of the power spectrum derived in this study, characteristic wavenumbers were determined to investigate the relationship between the wall-to-sheet distance and folding distance. Specifically, changes in the characteristic wavenumber resulting from changes in *l*_a_ and *l*_b_ were analyzed. Equation (13) provides the average wavenumber *u*^avg^ based on the power spectrum *I*(*x,y*), and *u*^avg^ was defined as the characteristic wavenumber:

13 $$\begin{array}{*{20}c} {u^{{{\text{avg}}}} = \frac{{\mathop \sum \nolimits_{x = 0}^{{\frac{{N_{x} }}{2}}} \mathop \sum \nolimits_{y = 0}^{{\frac{{N_{y} }}{2}}} \left( {\sqrt {u_{x}^{2} + u_{y}^{2} } I\left( {x, y} \right)} \right)}}{{\mathop \sum \nolimits_{x = 0}^{{\frac{{N_{x} }}{2}}} \mathop \sum \nolimits_{y = 0}^{{\frac{{N_{y} }}{2}}} I\left( {x, y} \right)}}.} \\ \end{array}$$

**Figure 4:**
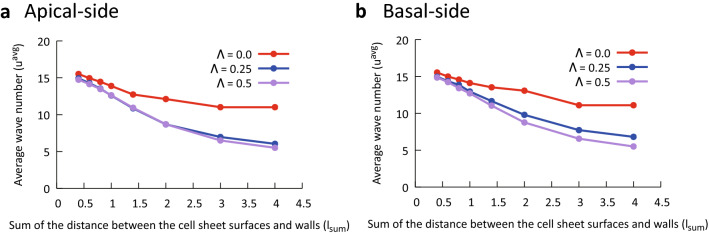
Relation between average wavenumber $u^{{{\text{avg}}}}$ and the sum of distances between the cell sheet surfaces and walls $l_{{{\text{sum}}}}$. The upper right legends show the degree of asymmetry ${\Lambda }$. (**a**) Apical-side results. (**b**) Basal-side results. The smaller the sum of distances between the sheet and walls, the smaller the average wavenumber of the folding. This is generally the case for the apical and basal sides.

Figure 4a graphically presents the apical-side results of the average wavenumber derived using the data described in the previous sections. Each point in Fig. 4 corresponds to a single simulation result. These results supported the finding that a smaller wall-to-sheet distance resulted in a smaller peak-to-peak folding distance. The wavenumbers on the basal side were determined similarly to those on the apical side, and the results are presented in Fig. 4b. The average wavenumbers on the apical and basal sides were generally comparable, with minor differences.

### Creating black-and-white images by binarizing Z-axis coordinate values

This section describes an indicator for the average longitudinal length of folds and provides a detailed quantitative analysis of its relationship with degree of asymmetry Λ. Here, the number of folds formed was chosen as a surrogate parameter inversely proportional to the total sum of the longitudinal lengths of the folds. We adopted this approach because determining the longitudinal length of the fold directly was not technically feasible. One major technical challenge was related to defining the fold’s ridge line, which is a key determinant of its longitudinal length. Another major reason for choosing this approach was that we used periodic boundary conditions in this study, and the longitudinal length of the fold was strongly dependent on the size of the simulation system. Consequently, the number of folds was determined as described below.

First, the area of computation was divided into unit grid cells of the same size, the total number of which was *N*_*x*_ × *N*_*y*_, where *N*_*x*_ and *N*_*y*_ denote the numbers of unit grid cells on the *x-* and *y-*axes, respectively (*N*_*x*_ = *N*_*y*_ = 35). A discrete second-order displacement field *z*(*x,y*) was derived by linking the displacements and coordinates (*x*, *y*) of the individual grid centers. For each unit grid cell, the *Z* value was compared with the threshold value $\frac{{\max \left( {z\left( {x,y} \right)} \right) + \min \left( {z\left( {x, y} \right)} \right)}}{2}$, and the unit grid cells whose *z* values were greater and smaller than the threshold were presented as white and black, respectively. The image processing library openCV was used to delineate and count the white and black areas.

**Figure 5:**
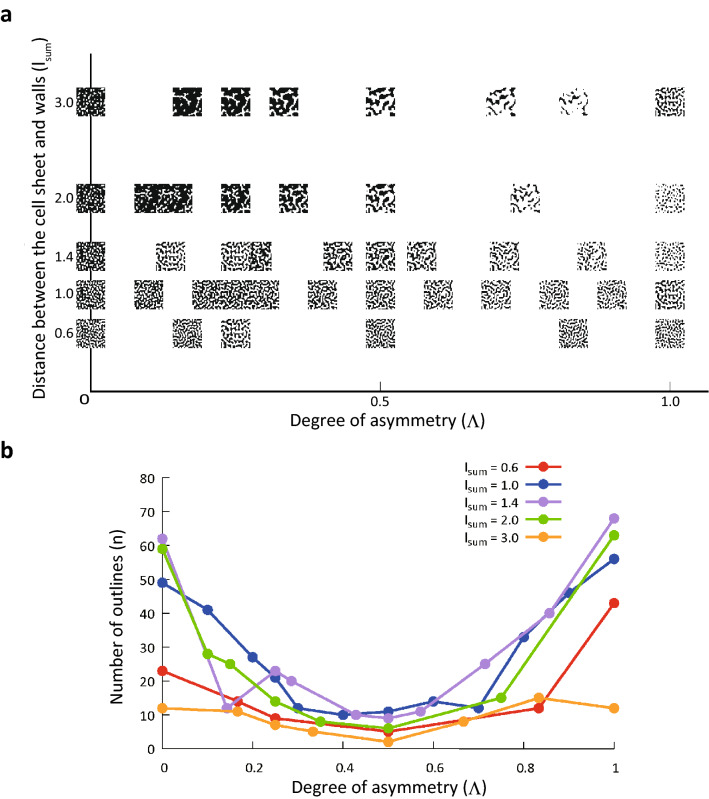
Binarized black-and-white images of the epithelial folding simulation results and the number of folds. (**a**) Relation between environment and folding pattern. The horizontal axis is the degree of asymmetry ${\Lambda }$. The vertical axis is the sum of distances between the cell sheet surfaces and walls $l_{{{\text{sum}}}}$. (**b**) Relation between degree of asymmetry ${\Lambda }$ and number of outlines. The upper right legends show the sum of the distances between the cell sheet surfaces and walls $l_{{{\text{sum}}}}$. The number of outlines $n$ was smallest when ${\Lambda }$ was 0.5, and $n$ increased as ${\Lambda }$ deviated from 0.5.

Figure 5a presents the binary black-and-white images obtained from the simulations described above, and Fig. 5b presents the number of folds identified based on the data presented in Fig. 3. The graph shows that the number of folds was the smallest at Λ = 0.5 for all simulation runs. In other words, increases in Λ from 0 to 0.5 (i.e., maximum asymmetry to symmetry) led to longer (and narrower) folds.

### Mechanical model derived from energy functional

In order to discuss the pattern formation depending on the degree of asymmetry Λ obtained from the 3D vertex simulation in terms of energy, we defined an energy functional of the cell sheet. Because buckling was caused by compression due to cell proliferation under periodic boundary conditions in the 3D vertex simulations, we considered the cell sheet compressed by force $N$ from $x,$ and $y$ directions. Based on the variational principle, the overdamped relaxation dynamics of the dimensionless displacement field $\tilde{w}(t,x,y$) is derived as follows.

14 $$\begin{array}{*{20}c} {\frac{{\partial \tilde{w}}}{{\partial \tilde{t}}} = - {\tilde{\Delta }}^{2} \tilde{w} - {\tilde{\Delta }}\tilde{w} - N\left( {\tilde{w}} \right)} \\ \end{array} ,$$

15 $$\begin{array}{*{20}c} {N\left( {\tilde{w}} \right) = \alpha \left( {R\left( {\tilde{w} - \left( {1 - {\Lambda }} \right)} \right) - R\left( { - \tilde{w} - {\Lambda }} \right)} \right)} \\ \end{array} ,$$

16 $$\begin{array}{*{20}c} {R\left( x \right) = \left\{ {\begin{array}{*{20}c} {x \left( {x \ge 0} \right)} \\ {0 (x < 0),} \\ \end{array} } \right.} \\ \end{array}$$

where ${\tilde{\Delta }}$ denotes the dimensionless Laplacian and $\alpha$ is a dimensionless constant. The term with the ramp function $R\left( x \right)$ comes from the fact that the stiffness of the elastic wall is expressed in terms of the step function in Eq. (8). The definition of the energy functional and details of the derivation of Eq. (14) are provided in the Supplementary Information.

**Figure 6:**
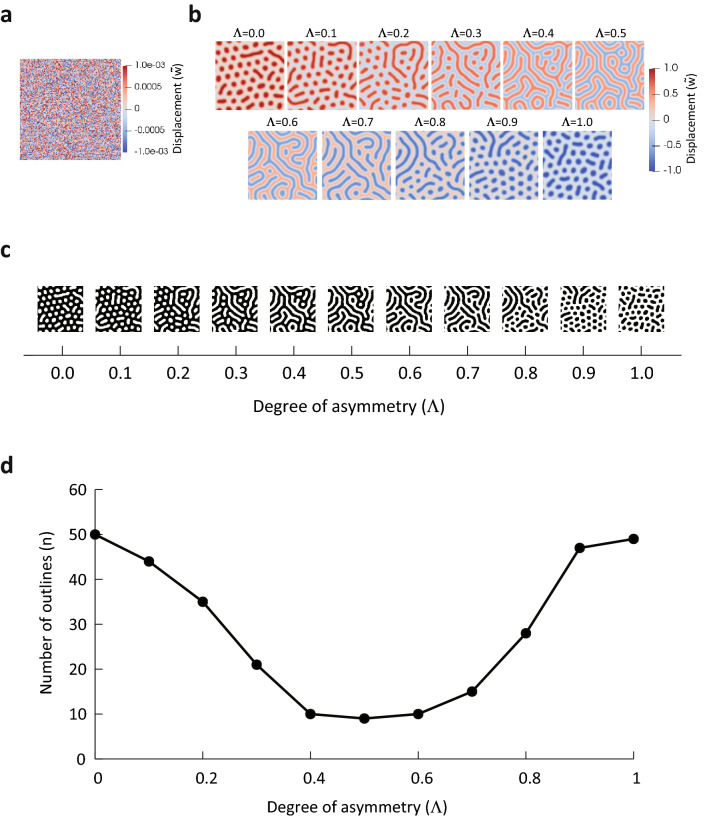
Simulation results for the mechanical model derived from the energy functional. (**a**) Initial state of displacement field $\tilde{w}$. (**b**) Snapshots of displacement field $\tilde{w}$ under the conditions of ${\Lambda }$ = 0.0 to 1.0 at time $t$ = 2,000,000. (**c**) Binarized black-and-white images of the simulation results. (**d**) Relation between degree of asymmetry ${\Lambda }$ and number of outlines.

We numerically integrated this differential equation with a second order Runge–Kutta scheme under periodic boundary conditions with ${\Delta }t = 1 \times 10^{ - 3} ,\, {\Delta }x = {\Delta }y = 0.6, \,N_{x} = N_{y} = 128, \,\alpha = 1000$. The initial state was set with randomly small displacements as shown in Fig. 6a. The displacement field $\tilde{w}$ at $t = 2,000,000$ is shown in Fig. 6b for varying the asymmetry degree Λ from 0.0 to 1.0. The results confirm a transition from a dot pattern to a hole pattern via a labyrinth pattern with increasing Λ as well as that observed in the 3D vertex simulation. Furthermore, to quantitatively evaluate this pattern transition, the displacement field was binarized (Fig. 6c) and the number of outlines was counted, as in the 3D vertex simulation. The results are shown in Fig. 6d. As it shows, the number of contours was large when Λ = 0.0, decreased closer to Λ = 0.5, and increased as Λ was further increased. This trend is comparable to the results of the 3D vertex simulation.

## Discussion

We examined the relationship between the epithelial folding patterns and the asymmetry of environmental constraints using the 3D vertex model, and in order to discuss the pattern formation depending on the degree of asymmetry Λ obtained from the 3D vertex simulation, we derived the mechanical model from the energy functional and confirmed that our derived equation could reproduce the results of the 3D vertex model.

The mechanical model with variational energy functional (Eq. 14) has a structure close to the Swift–Hohenberg (SH) equation of an order parameter $u$ as follows.

17 $$\begin{array}{*{20}c} {\frac{\partial u}{{\partial t}} = - a{\Delta }^{2} u - b\Delta u - ru + N\left( u \right)} \\ \end{array} .$$

According to a previous study^14^, the dynamics of a thin elastic film wrinkling on an elastic substrate is approximated by the SH equation using $N\left( u \right) = - cu^{3}$. The third and fourth terms on the right hand side (r.h.s.) of Eq. (16) are related to the energy of the substrate. This equation is known to reproduce pattern selection of hexagonal (dot pattern and hole pattern) and labyrinth patterns depending on $r$. Focusing on the first and second terms in r.h.s. of Eq. (16), substituting $\delta u = \sum\nolimits_{k} {c_{k} } e^{{\lambda_{k} + ikx + iky}}$ to the following equation:

18 $$\begin{array}{*{20}c} {\frac{\partial u}{{\partial \tilde{t}}} = - {\Delta }^{2} u - \Delta u .} \\ \end{array}$$

Examining the stability of the trivial solution $u = 0$, we obtain $\lambda_{k} = - k^{4} + k^{2}$, which means that there is the range of wave number in which order parameter $u$ is not damped, while the linear and cubic terms (third and fourth terms in r.h.s. of Eq. (16)) suppress the increase of order parameter $u$. In our mechanical model, the terms with the offset ramp functions in Eq. (15) are considered to play the role in suppression of the amplitude of $\tilde{w}$. Due to its strong nonlinearity term including offset ramp functions in our model, analytical solution of pattern selection is a future challenging work.

In this study, degree of asymmetry was the parameter for pattern selection. From a mathematical point of view, there are other phenomena of pattern formation that can be explained by the SH equation, and the parameters of pattern selection do not necessarily correspond to asymmetry. For example, in the system of spherically shaped elastic bilayer materials^14^, the parameter of pattern selection is the effective radius $R/h$, which is the ratio of the thickness $R$ of the substrate layer to the thickness $h$ of the film layer.

In summary, we investigated the relationship between epithelial folding pattern and environmental constraint on cell displacement using a 3D vertex model that describes morphological changes resulting from cell growth and division. The results revealed that the wall-to-sheet distance was a major determinant of the peak-to-peak folding distance. Furthermore, using a 3D vertex model and a mechanical model derived from the energy functional, numerical simulations showed that the degree of asymmetry with respect to the location of the upper and lower walls relative to the epithelial sheet resulted in different morphological patterns, such as dot patterns, labyrinth patterns, and hole patterns.

## Supplementary Information

Supplementary Video 1.

Supplementary Information.

## Data Availability

The datasets generated during and/or analyzed during the current study are available from the corresponding author on reasonable request.
