# Supplementary material for: Impact of environmental asymmetry on epithelial morphogenesis
Source: Sci Rep. 2022 Jul 5;12:11326. doi: 10.1038/s41598-022-15343-y (PMC9256595; doi:10.1038/s41598-022-15343-y)
Supplement: Supplementary file 2 — Supplementary Information. [file 41598_2022_15343_MOESM2_ESM.docx]

**Impact of Environmental Asymmetry on Epithelial Morphogenesis**

# **Kentaro Morikawa^*1^, Daichi Kuroda, and Yasuhiro Inoue^*2^**

Department of Micro Engineering, Graduate School of Engineering, Kyoto University, Kyoto, Japan

^*1^morikawa.kentaro.68a@kyoto-u.jp

^*2^inoue.yasuhiro.4n@kyoto-u.ac.jp

Supplementary Material

Supplementary Video 1 (ESV_1.mp4): Epithelial folding simulation under the condition sandwiched between two elastic walls. As cells proliferate under periodic boundary conditions, buckling of this tissue is induced. It causes the out-of-plane deformed epithelial tissue to collide with the wall ($l_{a}=l_{b}=0.5$).

Supplementary Information: definition of the energy functional and derivation of the equation (14)

**Supplementary Information: definition of energy functional and derivation of equation (14)**

We defined an energy functional of tissue sheet sandwiched by two elastic walls, one at a distance of $l_{a}$ and the other at a distance of $l_{b}$ from the neutral surface of the sheet. Let us set the $z$-axis so that the position of the apical wall is $z=l_{a}$ and the position of the basal wall is $z=-l_{b}$. Because buckling was caused by compression due to cell proliferation under periodic boundary conditions in the 3D vertex simulations, we consider a system compressed by force $N$from $x$ and $y$ directions. Then, the potential energy $U$ of the whole system is described by

$$U=\iint\left[ {\frac{1}{2}D\left( \Delta w \right)}^{2}-\frac{N}{2}\left( \nabla w \right)^{2}+\frac{1}{2}k_{0}\left( R\left( w-l_{a} \right) \right)^{2}+\frac{1}{2}k_{0}\left( R\left( -w-l_{b} \right) \right)^{2} \right]dxdy,$$

$$R\left( x \right)=\left\{ \begin{aligned} x (x\geq0) \\ 0 (x<0) \end{aligned} \right.$$

where $D$ is the bending stiffness and $k_{0}$ is the elastic modulus of the walls. The energy of the elastic wall is expressed using the ramp function $R(x)$, since it only has value when the displacement $w$ is $w<-l_{b}$ or $w>l_{a}$.

Taking a variation of $U$ with respect to $w$, we obtain the following:

$$-\frac{\delta U}{\delta w}=\iint\left[ -D\Delta^{2}w-N\Delta w-k_{0}\left( R\left( w-l_{a} \right)-R\left( -w-l_{b} \right) \right) \right]dxdy,$$

Thus, the overdamped relaxation dynamics of energy minimization in the displacement field $w$ can be written as follows:

$$\gamma\frac{\partial w}{\partial t}=-D\Delta^{2}w-N\Delta w-k_{0}\left( R\left( w-l_{a} \right)-R\left( -w-l_{b} \right) \right).$$

Let $\tilde{w}=\frac{w}{\mu}, \tilde{t}=\frac{t}{\tau}, \tilde{x}=\frac{x}{\lambda}, \tilde{y}=\frac{y}{\lambda}$, $\lambda=\sqrt{\frac{D}{N}}, \tau=\frac{\gamma D}{N^{2}}, \mu=l_{\mathrm{sum}}=l_{a}+l_{b}$, and using the definition of $\Lambda$ ($l_{a}=l_{\mathrm{sum}}\left( 1-\Lambda\right), l_{b}=l_{\mathrm{sum}}\Lambda$), we obtain the dimensionless equation

$$\frac{\partial\tilde{w}}{\partial\tilde{t}}=-\tilde{\Delta}^{2}\tilde{w}-\tilde{\Delta}\tilde{w}-\frac{k_{0}D}{N^{2}}\left( R\left( \tilde{w}-\left( 1-\Lambda\right) \right)-R\left( -\tilde{w}-\Lambda\right) \right).$$

Let $\alpha=\frac{k_{0}D}{N^{2}}$, we obtain equation (14).
